# The colonic flap: a versatile and reliable donor-site-free technique for pelvic reconstruction after exenteration

**DOI:** 10.3389/fonc.2025.1695995

**Published:** 2025-11-18

**Authors:** Chucheep Sahakitrungruang, Songphol Malakorn

**Affiliations:** 1 Chulalongkorn Colorectal Research Unit, Faculty of Medicine, Chulalongkorn University, Bangkok, Thailand; 2 Colorectal Surgery Division, Department of Surgery, Faculty of Medicine, Chulalongkorn University, Bangkok, Thailand

**Keywords:** colonic flap, pelvic reconstruction, pelvic exenteration, neovagina, rectal cancer, minimally invasive surgery, mucosa-removed flap

## Abstract

Pelvic exenteration for locally advanced or recurrent malignancies results in a large pelvic dead space and complex perineal defects, presenting formidable reconstructive challenges. Conventional methods, such as the vertical rectus abdominis myocutaneous (VRAM) flap, are effective but associated with significant donor-site morbidity and flap-specific complications. To overcome these limitations, the colonic flap was developed, a technique that utilizes a vascularized segment of sigmoid colon harvested within the operative field. This approach has two main applications: a mucosa-intact sigmoid flap for neovaginal reconstruction, and a mucosa-removed colonic flap for pelvic floor reconstruction and dead-space obliteration. This review outlines the limitations of existing techniques, the rationale for the colonic flap, its surgical principles, indications, clinical outcomes, and limitations. Based on more than a decade of clinical experience, the colonic flap has proven to be a safe, versatile, and effective reconstructive option. It avoids donor-site morbidity, is fully compatible with minimally invasive surgery, and provides excellent functional and oncologic outcomes. The colonic flap should be considered a valuable addition to reconstructive options in advanced pelvic surgery.

## 1 Introduction: the challenge of the empty pelvis

Radical resection procedures such as pelvic exenteration or extended abdominoperineal excision (APE) remain the only curative options for selected patients with locally advanced or recurrent pelvic malignancies (1). These operations create a large, non-collapsible pelvic dead space that predisposes to complications collectively described as the empty pelvis syndrome (2). These include wound dehiscence, pelvic abscesses, chronic sinuses, enteroperineal fistulas, adhesive small bowel obstruction, and perineal hernias (3). Radiation-induced tissue damage further exacerbates poor wound healing and infection risk.

Pelvic reconstruction with vascularized tissue is now standard to minimize morbidity. The goals are twofold: to obliterate pelvic dead space and, when required, to reconstruct resected organs such as the vagina. To address these challenges, we previously described the use of a colonic flap, a vascularized segment of sigmoid colon mobilized within the operative field, as a novel reconstructive option (4, 5). The initial reports demonstrated two major applications: the sigmoid flap with intact mucosa for neovaginal reconstruction (4), and the mucosa-removed colonic flap for pelvic floor reconstruction after exenteration (5). Creating a neo-pelvic floor with the colonic flap by positioning it low in the pelvis reduces dead space beneath the flap and increases abdominal volume which ultimately prevent the development of empty pelvis syndrome. In addition, the flap’s peritoneal serosal surface provides a natural barrier, significantly decreasing the risk of pelvic adhesion formation. Building on these foundations, this review summarizes the rationale, technical refinements, indications, and comparative analysis of the colonic flap with the other techniques for pelvic reconstruction.

## 2 Limitations of traditional reconstructive techniques

### 2.1 Pedicled myocutaneous flaps

Pedicled myocutaneous flaps remain the cornerstone of pelvic reconstruction, particularly the VRAM flap, gracilis flap, and gluteal flap (6–8). While effective, these approaches have significant drawbacks:

Donor-site morbidity: VRAM harvest weakens the abdominal wall and increases the risk of incisional or parastomal hernias, with reported rates ranging from 10% to 35% (9, 10). Other complications include wound dehiscence, infection, and chronic pain. This is especially concerning for patients requiring permanent stomas.
Flap-specific complications: Rates of partial or complete flap necrosis requiring re-intervention are well documented, occurring in 5% to 20% of cases depending on the series and flap type (10, 11).
Functional limitations in neovaginal reconstruction: Keratinized skin flaps often result in dryness, stenosis, discharge, and discomfort, compromising sexual function (12).
Incompatibility with minimally invasive surgery: Large incisions required for flap harvest undermine the benefits of laparoscopic or robotic resection.

### 2.2 Mesh reconstruction

The use of biologic or synthetic mesh to bridge the pelvic inlet has been proposed as a technically simpler alternative to autologous flap reconstruction (13). Biologic meshes, such as acellular dermal matrices, were initially favored for their presumed resistance to infection; however, clinical reports have documented notable complications, including seroma formation in approximately 8%, transient perineal pain in 33% (14), and infection rates of up to 17% (15). Evidence regarding postoperative bulging or herniation remains inconsistent, with some studies reporting no herniation following biologic mesh repair (15). While minimally invasive approaches are technically feasible, they remain inadequate for reconstructing a neovagina or addressing extensive perineal skin defects. Mesh may serve as reinforcement when combined with autologous tissue, yet its role as a standalone solution continues to be debated. These limitations underscore the need for a reliable autologous alternative that avoids donor-site morbidity.

## 3 The colonic flap: rationale and innovation

The colonic flap was designed to address the shortcomings of conventional methods by utilizing sigmoid colon tissue, which is typically mobilized during pelvic resection. Supplied by preserved sigmoid vessels after low ligation of the inferior mesenteric artery, the flap provides robust vascularity without additional incisions. Two configurations have been developed:

### 3.1 Sigmoid flap with intact mucosa (neovaginal reconstruction)

**Figure 1:**
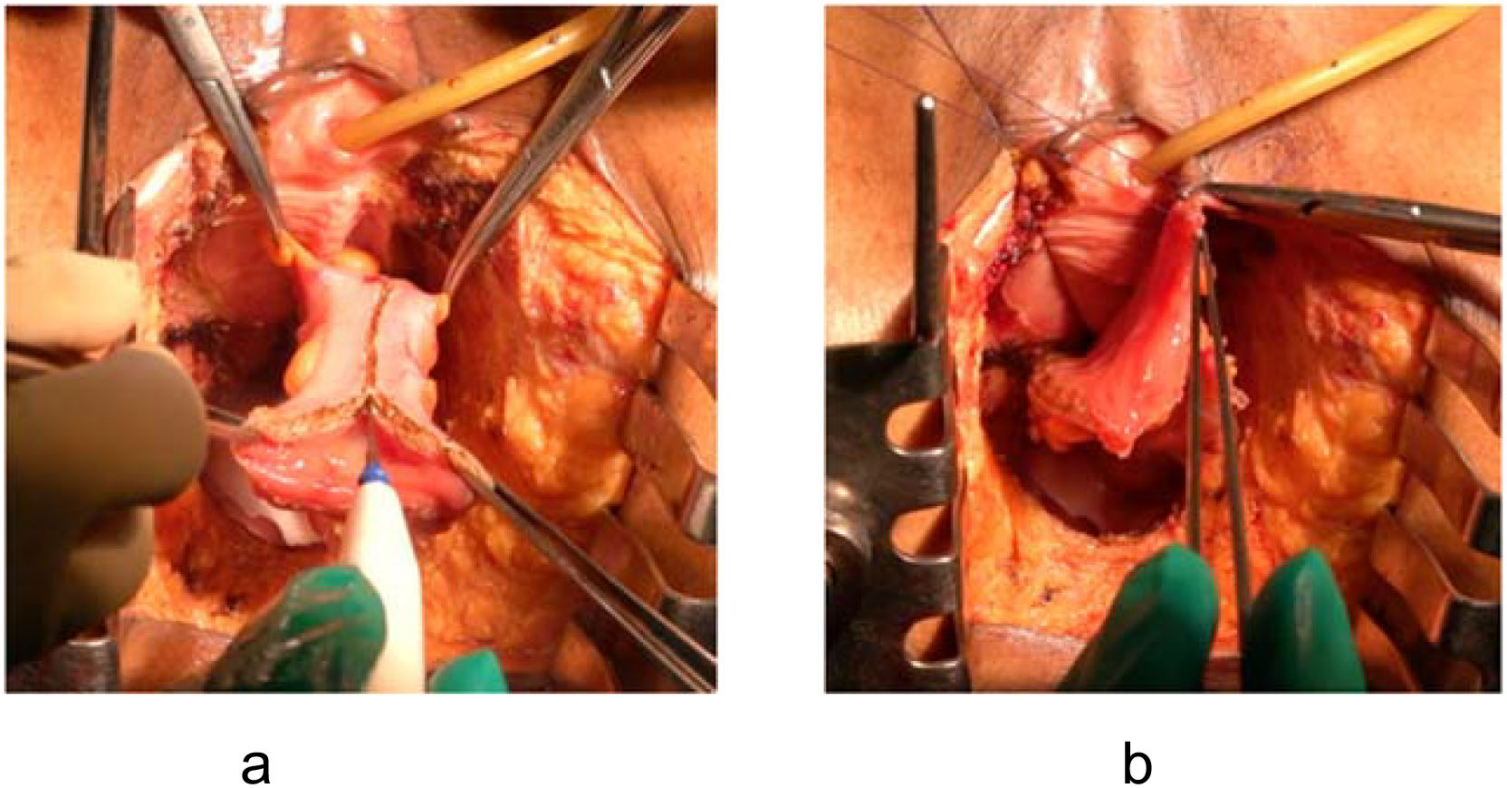
Mucosa-intact colonic flap for neovaginal reconstruction. **(a)** Spatulation of the sigmoid colon along the anti-mesenteric border. **(b)** Anastomosis of the flap to the remnant anterior vaginal wall, forming a self-lubricating neovagina.

For female patients undergoing APE with en bloc vaginectomy, the mucosa-intact sigmoid flap mimics the natural vaginal environment (**Figure 1**).

Advantages: The colonic mucosa provides self-lubrication, maintains luminal width and depth, and avoids the dryness, keratinization, and irritation seen with skin flaps (4). This translates into improved sexual function and patient satisfaction.

### 3.2 Mucosa-removed colonic flap (pelvic floor reconstruction)

**Figure 2:**
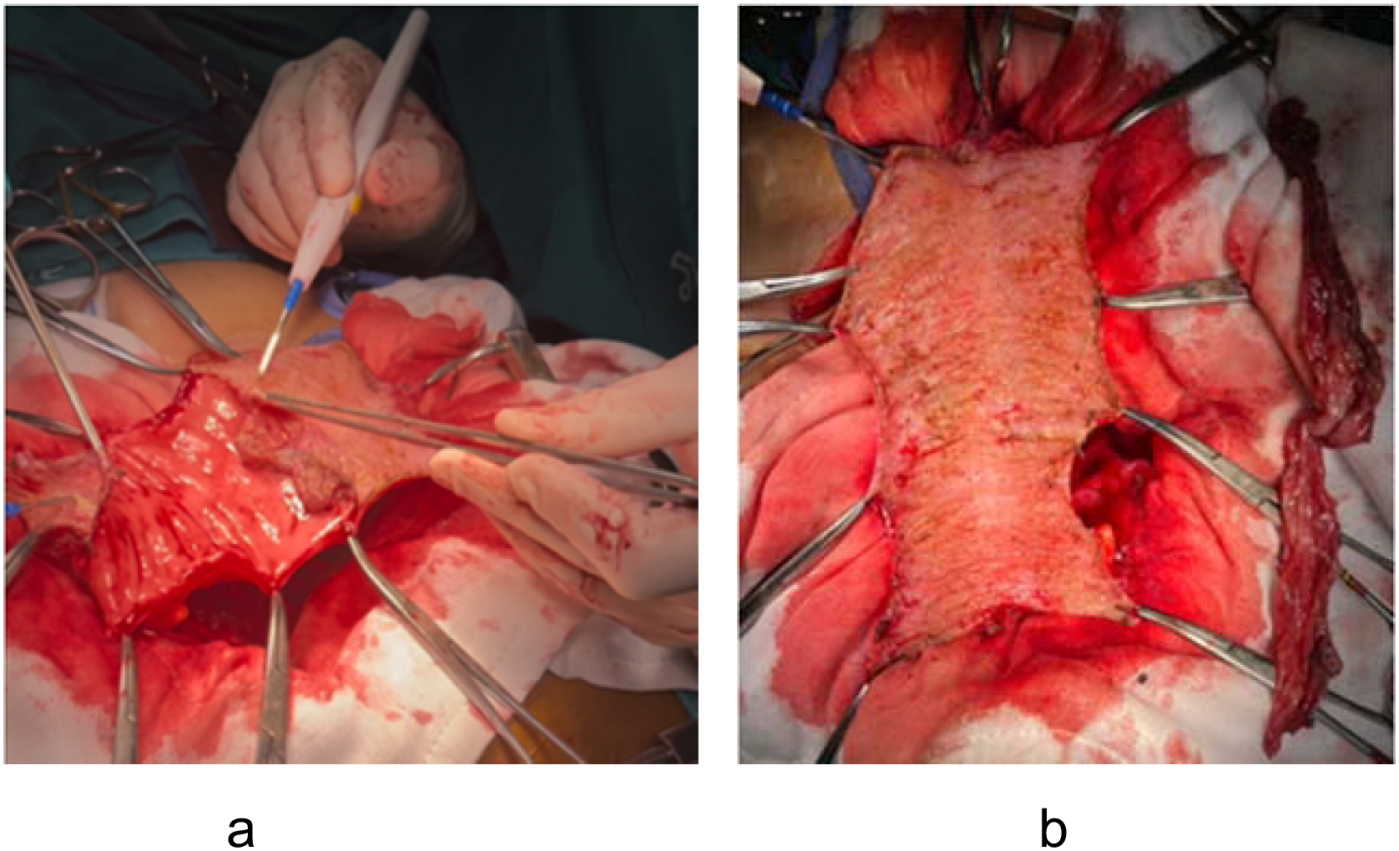
Mucosa-removed colonic flap for pelvic floor reconstruction. **(a)** Mucosectomy performed using electrocautery to remove the colonic mucosa. **(b)** Resulting seromuscular flap prepared for pelvic placement.

For cases requiring only pelvic floor reconstruction, the mucosa is unnecessary and its secretion is undesirable (**Figure 2**).

Advantages: Submucosal dissection (mucosectomy) converts the colonic segment into a vascularized seromuscular flap (5). This flap reconstructs a new pelvic floor, prevents perineal hernia, and may reduce small bowel adhesions due to the peritonealized surface of the flap. Furthermore, the potential pelvic dead space is effectively eliminated when the flap is placed downward to the pelvic floor.

Thus, the colonic flap provides a versatile, anatomically compatible solution for two distinct reconstructive needs: neovaginal creation and pelvic floor restoration.

## 4 Surgical technique and application

The procedure is integrated into the primary oncologic resection.

**Figure 3:**
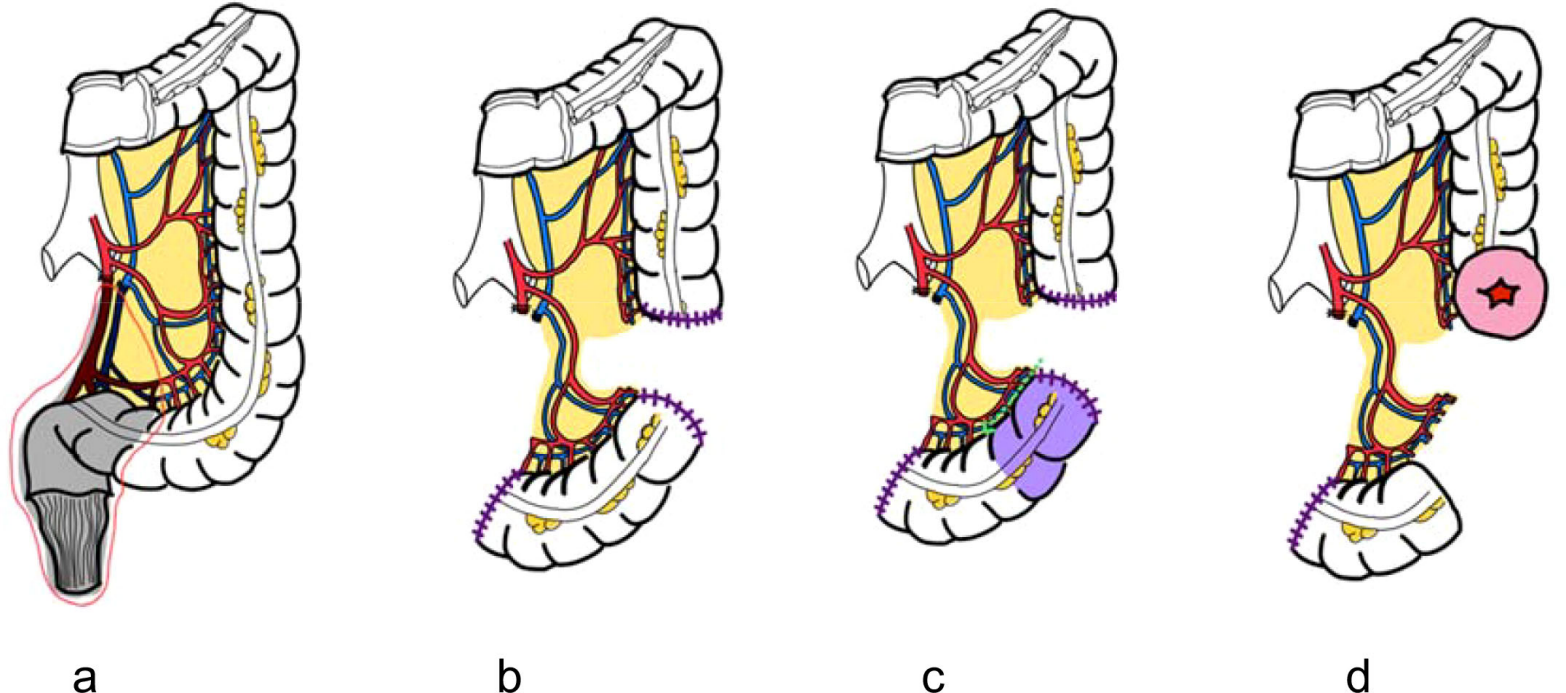
Schematic illustration of colonic flap harvest technique. **(a)** Low ligation of the inferior mesenteric artery to preserve sigmoid vascular supply. **(b)** Mesenteric division with preservation of arterial and venous drainage via the inferior mesenteric vein (IMV). **(c)** Resection of the proximal segment with careful dissection near the colonic wall to maintain marginal vessels. **(d)** Creation of proximal colostomy and harvest of a well-vascularized colonic flap.

**Figure 4:**
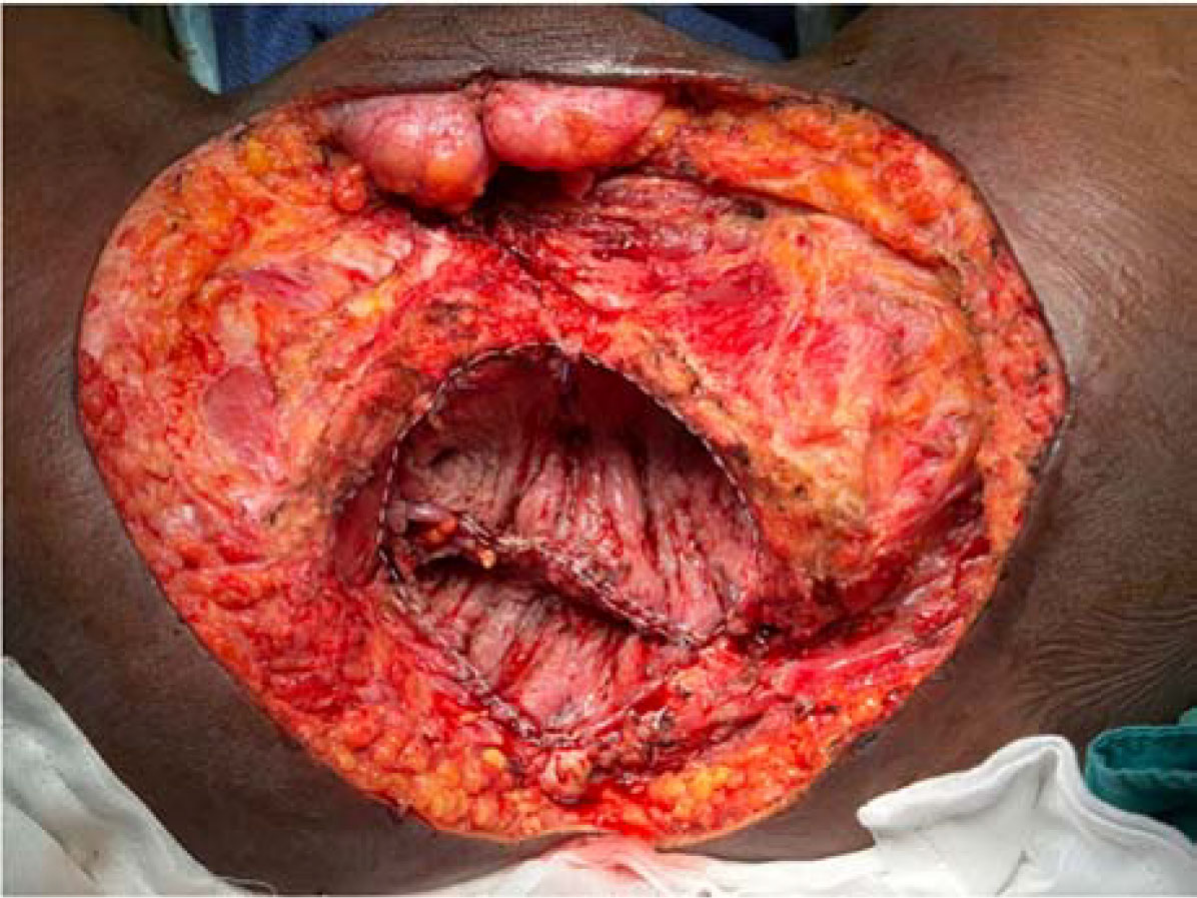
U-shaped configuration of mucosa-removed colonic flap. The dotted outline illustrates flap design tailored to cover a large pelvic defect following total pelvic exenteration.

**Figure 5:**
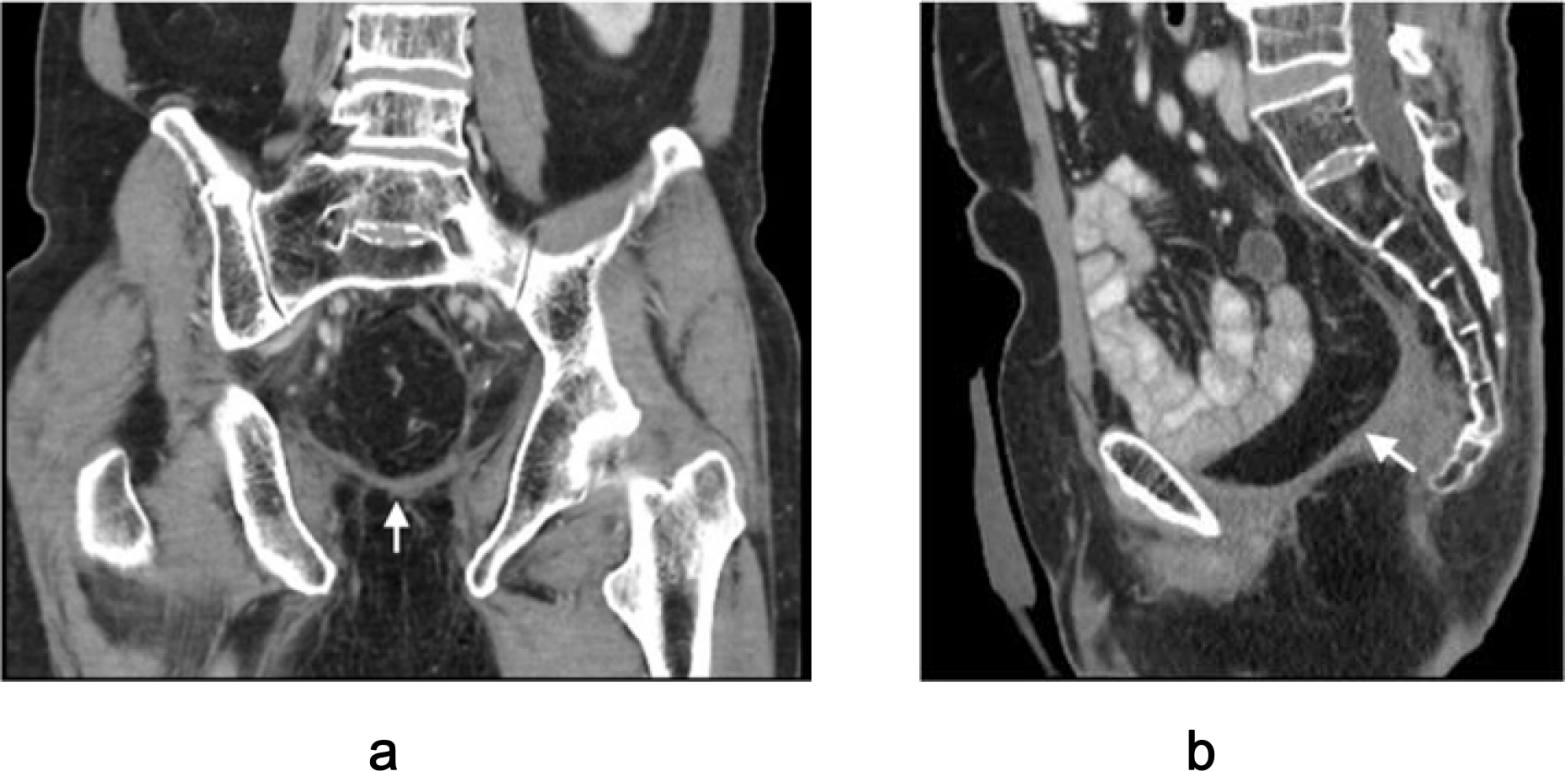
Postoperative CT imaging of mucosa-removed colonic flap. **(a)** Coronal view; **(b)** Sagittal view. Arrows indicate optimal alignment of the flap forming a neoperitoneal diaphragm, effectively preventing perineal herniation.

Flap harvest: A sigmoid segment is mobilized based on preserved sigmoid vessels. A critical point is the meticulous preservation of the vascular pedicle, ensuring not only the arterial supply but also the venous drainage to the inferior mesenteric vein (IMV) which is paramount to prevent venous congestion of the flap. To ensure the flap reaches the deep pelvis without tension, the most distal portion of the mobilized sigmoid is utilized for the reconstruction (**Figure 3**).
Preparation: The preparation of the flap differs based on its intended application.
For neovaginal reconstruction: The flap is spatulated along its anti-mesenteric border. The length of the mucosa-intact colonic flap should not be longer than 10 cm to minimize excess mucous production and ease subsequent neovaginal care. If a longer segment is initially mobilized to provide adequate reach, the proximal part of the flap is removed. This devascularized segment is resected by performing a dissection close to the colonic wall to ensure the marginal vessels supplying the final flap are preserved.
For pelvic floor reconstruction: The flap is first spatulated and then a mucosectomy is carefully performed using electrocautery. The idea for flap elongation is the same, however the length of the flap can be designed according to the size of the defect. To address a large pelvic defect such as total pelvic exenteration with sacrectomy, the flap can be designed up to 30 cm in length. This long, mucosa-removed flap can then be stitched together into a U-shape configuration to effectively cover the large pelvic defect (**Figure 4**).
Placement: The flap is positioned without tension. Neovaginas are sutured to the anterior vaginal remnant; mucosa-removed flaps are anchored to the pelvic sidewalls and presacral fascia to recreate a peritoneal diaphragm with the peritonealized serosal surface facing toward the abdominal cavity (**Figure 5**).

A key advantage is seamless integration with minimally invasive surgery. The technique’s utility has been demonstrated in increasingly complex scenarios, including laparoscopic pelvic exenteration requiring neovagina or pelvic floor reconstruction (16–18).

Intraoperative flap failure represents the most significant limitation. In the authors’ experience, this typically results from either high ligation of the inferior mesenteric artery during prior surgery or an inherently short sigmoid mesentery, both of which can prevent the flap from reaching the pelvic floor without tension. This challenge can be addressed by fully mobilizing the splenic flexure to provide adequate reach. Colon transection should be performed at a point where the arterial and venous supply of the vascular pedicle remains robust. The most distal portion of the flap is typically used for reconstruction, while the unused proximal colon is carefully separated from the mesentery, with meticulous preservation of the marginal vessels. In cases where the sigmoid segment remains unsuitable following these maneuvers, the cecal flap based on the ileocolic vessels serves as a reliable alternative.

**Figure 6:**
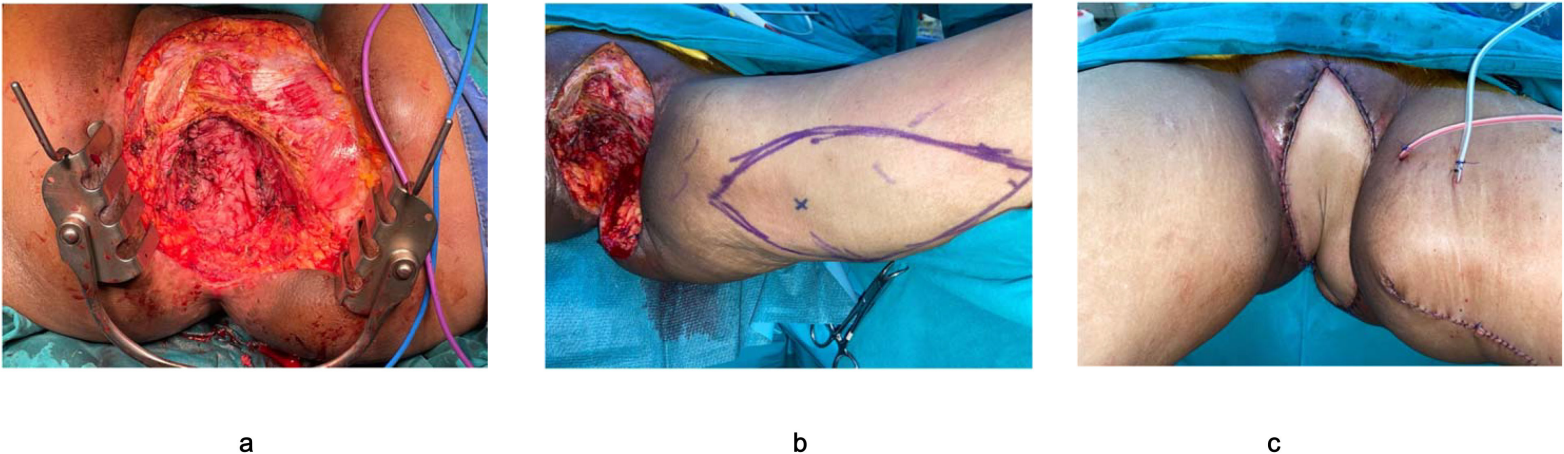
Reconstruction after pelvic exenteration with large perineal resection. **(a)** Placement of mucosa-removed colonic flap as neopelvic floor. **(b)** Pedicular myocutaneous flap planned from the patient’s thigh. **(c)** Reconstruction of large perineal defect with a well-vacularized myocutaneous flap.

## 5 Comparative analysis and literature synthesis

The choice of reconstructive technique involves a trade-off between reconstructive goals and procedure-related morbidity. **Table 1** synthesizes the literature, comparing the main reconstructive options. This comparison highlights a fundamental divide. The VRAM flap provides excellent vascularity but at the cost of high donor-site morbidity and notable rates of recipient-site complications like partial flap necrosis and functional deficits. Mesh reconstruction eliminates donor-site morbidity but trades it for a high risk of failure at the recipient site. The colonic flap occupies a unique position by eliminating donor-site morbidity while simultaneously minimizing recipient-site complications. Its excellent, surgically preserved vascularity makes ischemia rare, and its inherent tissue properties provide superior functional outcomes for neovaginal reconstruction and durable prevention of perineal hernias. Moreover, a minimally invasive approach can be utilized.

**Table 1:** Comparative analysis of various techniques for pelvic reconstruction.

Technique	Vascularity	Infection risk	Flap bulk	Donor-Site morbidity	Neovagina reconstruction	Pelvic floor reconstruction	Large perineal defect reconstruction	MIS compatibility
VRAM	Excellent	Moderate	High	High	Good	Good	Good	Low
Gracilis	Variable	Moderate	Low	Moderate	Poor	Poor	Poor (small tissue bulk)	Moderate
Mesh	None	High	None	None	Cannot use	Good	No (Need myocutaneous flap)	High
Colonic Flap	Excellent	Low	Moderate	None	Excellent	Excellent	No (Need myocutaneous flap)	High

## 6 Discussion

Pelvic reconstruction after radical oncologic surgery must address two core problems: (1) obliteration of the pelvic dead space to prevent complications of the empty pelvis syndrome, and (2) restoration of organ-specific function, such as neovagina creation, when indicated (2, 3). The proper choice of reconstructive technique remains under debate. Advocates of myocutaneous flaps such as the VRAM, gracilis, and gluteus maximus argue that these provide reliable vascularity and sufficient bulk for dead-space obliteration (6–8), but skin-based neovaginas frequently develop dryness, stenosis, and poor functional outcomes (12). Moreover, concerns persist regarding donor-site morbidity, including abdominal wall weakness, parastomal hernia, and wound complications, with reported hernia rates as high as 35% (9, 10). Opponents of mesh-based repairs point to high failure rates, especially in irradiated fields, where biologic or synthetic meshes are associated with infection, seroma, and herniation (13–15).

The colonic flap has been proposed as a donor-site–free alternative that avoids these complications while maintaining robust vascularity (4, 5). Its proponents highlight favorable functional outcomes, particularly in neovaginal reconstruction, where colonic mucosa provides lubrication and elasticity superior to skin flaps (12). For pelvic floor reconstruction, the mucosa-removed colonic flap functions as a vascularized seromuscular layer, reducing perineal hernia and potentially limiting small-bowel adhesions through its peritonealized surface (5). Apart from all unique benefits, this technique can be applied in the minimally invasive setting (16–18). Nevertheless, critics note that current evidence is based primarily on single-center case series (4, 5, 16–18), raising questions about reproducibility and generalizability across diverse surgical practices.

### 6.1 Current research gaps and future perspectives

Despite encouraging outcomes, several gaps remain. Most published studies are retrospective, involve small sample sizes, and lack standardized outcome reporting (4, 5, 16–18). Comparative analyses with VRAM, gracilis, or mesh reconstructions are sparse, limiting direct evidence of superiority (6–15). Furthermore, long-term oncologic outcomes and quality-of-life data, especially sexual function following neovaginal reconstruction, remain underreported (12). Cost-effectiveness studies are also lacking, despite increasing attention to health economics in complex oncologic surgery. Addressing these evidence gaps through multicenter prospective studies or registry-based data collection will be essential to define the true role of the colonic flap in contemporary practice.

Several avenues for future development can be anticipated. First, the wider application of minimally invasive and robotic surgery is likely to expand the indications for the colonic flap, given its compatibility with laparoscopic harvest and placement (16–18). Second, multicenter collaborations and standardized reporting frameworks—similar to those established by the PelvEx Collaborative (2)—could provide higher-quality data to benchmark outcomes and refine patient selection criteria. Third, as functional recovery and survivorship become increasingly central in oncologic care, studies specifically addressing long-term quality of life, sexual health, and body image will be necessary to fully evaluate reconstructive success. Finally, integration of the colonic flap into combined strategies (e.g., colonic flap for pelvic floor with adjunctive skin flaps for large external perineal defects) represents a pragmatic pathway to tailor reconstruction to individual patient needs.

### 6.2 Considerations

Appropriate patient selection and technical awareness are critical for the success of the colonic flap.

Patient selection: The technique may not be suitable for all patients. A history of extensive prior colonic surgery, significant adhesions, or active inflammatory bowel disease involving the sigmoid colon are relative contraindications. However, patients with diverticular disease may still be suitable candidates if the selected colonic segment appears healthy and well vascularized. During mucosectomy, all mucosa must be meticulously removed. In patients with diverticulosis, the procedure can still be performed successfully; although small openings may occur in the muscular layer of the colonic flap, the integrity and viability of the flap remain preserved.
Scope of reconstruction: The colonic flap is designed for internal reconstruction of the pelvis and/or vagina. It does not provide a skin paddle and is therefore unsuitable for cases requiring large-scale external perineal skin closure. In such scenarios, the colonic flap is still the flap of choice to reconstruct the pelvic floor and a myocutaneous flap can be added on to address the large defect below the colonic flap. (**Figure 6**).
Technical demands: While the procedure is conceptually straightforward, it does increase overall operative time and requires meticulous vascular dissection to ensure flap viability. However, the learning curve is relatively short, as most colorectal surgeons are already familiar with colonic mobilization techniques.

### 6.3 Limitations

This review is narrative in nature. Relevant studies were identified through PubMed using combinations of the terms *pelvic reconstruction*, *colonic flap*, *VRAM*, *mesh*, and *exenteration* up to September 2025. Most available data on the colonic flap derive from single-center, retrospective experiences. Articles were selected for their clinical relevance and applicability rather than through a systematic inclusion process.

## 7 Conclusion

Pelvic reconstruction is a critical component of optimizing outcomes after radical oncologic surgery. The colonic flap, in both its mucosa-intact and mucosa-removed forms, offers a versatile, safe, and donor-site–free alternative to conventional flaps and mesh repair. In the authors’ experience, it seems to provide superior functional outcomes in neovaginal reconstruction, reliable reinforcement of the pelvic floor with effective dead-space obliteration, and seamless integration with minimally invasive approaches. Importantly, the harvest of the colonic flap is technically simple and familiar to colorectal surgeons, making it a practical and reproducible option for routine clinical use. Therefore, the colonic flap represents a paradigm shift in reconstructive pelvic surgery and should be regarded as a valuable addition to the surgical oncologist’s armamentarium.
